# Editorial: Music therapy in geriatrics, volume II

**DOI:** 10.3389/fmed.2024.1383160

**Published:** 2024-02-29

**Authors:** Suzanne B. Hanser, Amy Clements-Cortés, Melissa Mercadal-Brotons, Concetta Maria Tomaino

**Affiliations:** ^1^Music Therapy Department, Berklee College of Music, Boston, MA, United States; ^2^Faculty of Music, University of Toronto, Toronto, ON, Canada; ^3^Catalonia College of Music, Barcelona, Spain; ^4^Institute for Music and Neurologic Function, New York, NY, United States

**Keywords:** music therapy, music-based interventions, dementia, community, caregivers, older adults

The World Health Organization (WHO) has called attention to the escalating evolution of the aging population such that the number of people over 60 years of age will almost double between 2015 and 2050, representing a change of 12%−22% of the global population. WHO has reported that in the year 2020, the number of people in the world over 60 exceeded the number of people under 5. In response, the United Nations Decade of Healthy Aging (2021–2030) has brought with it an international plan of action, in conjunction with the United Nations Agenda 2030 on Sustainable Development and the Sustainable Development Goals (1). This strategy emphasizes a positive approach to community development that nurtures the abilities of older adults, provides services that address their needs, modifies attitudes of ageism, and promotes accessibility to long-term care, while narrowing gaps of health disparities.

In conjunction with this mission, WHO established an Arts and Health Initiative, under the leadership of Christopher Bailey, in recognition of the ability of the arts to promote health, help manage health conditions, and prevent disease (2). The establishment of this initiative is an important step in heightening awareness of the many significant clinical benefits of music and the arts.

In the United States, “Sound Health,” a partnership between the National Institutes of Health (NIH), the Kennedy Center for Performing Arts, and the National Endowment for the Arts, has established a richly-funded research agenda, encompassing basic and mechanistic research, translational and clinical research, methods and outcomes, and capacity-building and infrastructure. The Sound Health Network accompanies this research effort with a web of resources to inform the public about the wide-ranging impact of music, and how it can be applied for health and wellness, as well as improvements for those with neurological conditions (3).

There is a growing body of scientific evidence to support music-based interventions that enhance brain health, emotional and social wellbeing, and even, cognitive improvement (4). The Global Council on Brain Health has articulated the benefits and potential of music to “promote brain health and mental wellbeing” in their report on the subject. They also included practical recommendations for music engagement as well as music-based interventions. It is clear that the potential of the arts is being acknowledged around the world and resources are being devoted to aid implementation and dissemination of empirical findings (5).

The number of people living with dementia has been estimated to be over 55 million and growing rapidly worldwide. Without a cure for Alzheimer's disease or related disorders causing dementia, and lacking treatments for the decline in the associated neurological functioning, the enormous burden and costs to individuals, family, community, and society at large will only increase over time (6).

In its effort to foster rigorous research designs that demonstrate efficacy and effectiveness of music-based interventions, NIH developed guidelines for research on music in addressing brain disorders of aging. With the assistance of experts in multiple disciplines, the NIH created a toolkit to guide research agenda and provide healthcare providers with advice regarding how to prescribe music therapy services (7).

Given the burgeoning research and positive findings regarding the impact of music therapy on older adults, the editors of the Research Topic, “Music Therapy in Geriatrics – Volume I,” were asked to update and expand the offerings in this new Volume II. This current Research Topic contains articles from Australia, Denmark, Germany, Israel, Korea, Netherlands, Norway, UK, and the states of Minnesota, Alabama, North Carolina, and Arizona in the USA.

Three contributions address specific interventions for older adults living in the community. Park et al. describe a music-based exercise training program designed for older adults who are experiencing mild-to-moderate cognitive decline. In their feasibility study, the investigators examined how rhythmic auditory stimulation and other strategies can motivate older adults to comply with exercise programs, and ultimately benefit from this multicomponent approach to exercise.

Gilboa and Levy demonstrate the impact of creating musical life reviews with community-dwelling older adults. Their case study and thematic analysis demonstrates the many ways that the process improved functional ability of participants, but perhaps most importantly, provided a very meaningful and enjoyable way to honor their life experiences.

Belgrave et al. discuss some very creative programming crafted during the isolation of the pandemic and provided virtually. A collaboration between a music therapy program and a global music museum forged an asynchronous offering to enhance overall wellness and memory care. The program enhanced engagement and sense of wellbeing.

One age-related condition which has perhaps achieved less attention than other disorders is presbyphagia, a disorder of the swallowing mechanism. Kim et al. offer a scoping review of music-based interventions, and report on the impact of activities like breathing as preparation for singing, and the training of singing with emphasis on controlling the larynx and oral-motor connections.

Two articles are investigations of the influence of music on people with dementia.

In their study, Prick et al. report the findings of a randomized controlled trial that assessed the impact of two interventions, namely active individual music therapy and individual music listening, on neuropsychiatric symptoms and quality of life in individuals with dementia residing in nursing homes. Additionally, the study examined the effect of these interventions on the burden experienced by professional caregivers, comparing them to a control group. The authors, drawing from their results, assert that there is no significant superiority of one intervention over the other. Consequently, they recommend the consideration of both interventions in clinical practice.

Wang et al. present a rationale for implementing a protocolized music teletherapy as a psychosocial intervention to mitigate neuropsychiatric symptoms in individuals with dementia. Their aim is also to enhance accessibility to music therapy for this population. The proposal is grounded in an extensive review of the current literature on music therapy and Alzheimer's disease.

Four articles reflect the need to educate both professional and family caregivers of people with dementia. Three of these describe programs for spouses and families.

McMahon et al. present the outcomes of a HOMESIDE sub-study involving six dyads, each comprising an individual with dementia and a family caregiver. These dyads participated in a 12-week home-based skill-sharing music intervention facilitated by a music therapist. The results, derived from thematic analysis of interviews and participant diaries, underscore the significance of personalized interventions and the therapeutic relationship. The findings reveal the efficacy of personalized music interventions as dyads learned to harness the therapeutic potential of music.

Thompson et al. share the development of the Music Attuned Technology-Care via eHealth (MATCH) mobile application created to train family caregivers on how to support persons with dementia through music engagement. In their content and validation study 10 music therapists with specialties in dementia care and seven family caregivers who had participated in previous training in the HOMESIDE project assessed the app's training modules. The content was seen as beneficial, and with suggestions, the MATCH application will be further adapted and explored in research with family caregivers and persons living with dementia.

Rosenbach et al. share the results of their multiple qualitative case study on a home-based music therapy (HBMT) model that combined weekly joint music therapy sessions and bi-weekly phone counseling sessions with the primary caregivers. Results indicated joint music therapy sessions strengthened the couple's relationships, practicing music together was essential for subsequent use by the personal caregivers, and three other themes point to the importance of the support provided.

Lastly, Ridder et al. detail the process of writing a training manual for music therapists to use when providing guidance to professional caregivers of people with late stage dementia on how to implement person-attuned musical interactions (PAMI). Their manual is the outcome of research that explored how music therapists collaborating with carers can support non-verbal communication in residential care settings.

In conclusion, the studies included in the Research Topic on *Music Therapy in Geriatircs, Vol II* provide further evidence of the efficacy of music therapy and music- based interventions in improving quality of life for those with Alzheimer's disease and related dementias as well as the ease of caring for their care partners. Several studies highlight the impact of training care partners in the use of music-based interventions to enhance interpersonal relationships and reduce social isolation that often occurs in those with neurocognitive deficits. Included too are informative guidelines on implementing music-based interventions. By including evidence based research from international experts, this volume will expand the knowledge of best practices of music therapy in geriatric care.

## Author contributions

SH: Conceptualization, Methodology, Project administration, Resources, Writing – original draft, Writing – review & editing. AC-C: Resources, Writing – original draft, Writing – review & editing. MM-B: Resources, Writing – original draft, Writing – review & editing. CT: Writing – original draft, Writing – review & editing.
